# The complete chloroplast genome of the *Crataegus kansuensis* (Rosaceae): characterization and phylogeny

**DOI:** 10.1080/23802359.2020.1792368

**Published:** 2020-07-20

**Authors:** Xiaobo Zhang, Yiheng Wang, Mengli Wang, Qingjun Yuan, Luqi Huang

**Affiliations:** National Resource Center for Chinese Meteria Medica, China Academy of Chinese Medical Sciences, Beijing, China

**Keywords:** *Crataegus kansuensis*, chloroplast genome, phylogenetic analysis

## Abstract

*Crataegus kansuensis* Wils. is an important wild eco economical species of the family Rosaceae. The complete chloroplast genome reported here is 159,865 bp in length, including two inverted repeats (IRs) of 26,384 bp, which are separated by a large single-copy (LSC) and a small single-copy (SSC) of 87,815 bp and 19,282 bp, respectively. The whole chloroplast genome of *C. kansuensis* contains 113 genes, including 79 protein-coding genes, 30 transfer RNA, and 4 ribosome RNA. Phylogenetic analysis indicated that *C. kansuensis* is closely related to that of *C. chungtienensis* and *C. marshallii*, and the genus *Crataegus* L. was sister to the genus *Amelanchier* Medik.

Gansu hawthorn (*Crataegus kansuensis* E.H.Wilson) is a fruit tree within the family Rosaceae, and is naturally distributed in the west and northwest of China. This species has very important ecological and economic values in the ecosystem of the shrub forest community. Hawthorn fruits are edible and used in traditional medicine, and there is a sizable market for natural health products (NHPs) made from hawthorn leaves, flowers, and fruits (Edwards et al. 2012). However, the genus *Crataegus* L. has long been recognized as one of the taxonomically challenging group due to the problems evoked by polyploidy, hybridization, and apomixis. In the present study, we determined the complete chloroplast genome (cpDNA) sequence of the Gansu hawthorn based on the next-generation sequencing method. The annotated cpDNA has been deposited into GenBank with the accession number MF784433. It is necessary to develop genomic resources for *C. kansuensis* for its utilization and to provide valuable genetic information for the phylogenetic studies.

We extracted the total genomic DNA from the fresh leaves of a single individual using the method of Li et al. (2013). And the sequencing library was constructed and quantified following the methods introduced by Dong et al. (2017). DNA sample and voucher specimen of *C*. *kansuensis* were deposited at the herbarium of the Institute of Chinese Materia Medica (Specimen accession number: PGP00008), China Academy of Chinese Medical Sciences (N39°56′18.96′′, E116°25′38.64′′). The whole-genome sequencing was conducted with 150 bp paired-end reads on the Illumina HiSeq X Ten platform. In all, 3.78 G raw reads were obtained, and after the quality-trimmed using the software CLC Genomics Workbench v7.5 (CLC bio, Aarhus, Denmark), the resultant 3.77 G reads were assembled with the program SPAdes 3.6.1 (Bankevich et al. 2012) (*Kmer* = 95). The chloroplast genome contigs selected by the Blast program, taken *Pyrus pyrifolia* (GenBank: AP012207) as the reference. The selected contigs were assembled using Sequencher 4.10. Gene annotation of *C. kansuensis* was performed using DOGMA annotation (Wyman et al. 2004) and manually corrected for codons and gene boundaries using BLAST searches.

The circular cpDNA of *C*. *kansuensis* was 159,865 bp in length, containing two short inverted repeat (IRa and IRb) regions of 26,384 bp, each, which was separated by a large single-copy (LSC) region of 87,815 bp and a small single-copy (SSC) region of 19,282 bp. The GC content of the whole chloroplast genome was 36.6%. The cpDNA of the Gansu hawthorn comprised 113 genes, including 79 protein-coding genes, 4 ribosomal RNA genes, and 30 transfer RNA genes. In these genes, 19 were duplicated in the IR regions and 19 genes contained one or two introns. 17 harbored a single intron, and 2 (*ycf3* and *clpP*) contained double introns.

A phylogenetic tree was constructed to confirm the location of *C*. *kansuensis* (Figure 1). We used 77 genes within the family Rosaceae to conduct a maximum-likelihood (ML) analysis using IQ-tree with 1000 bootstrap replicates (Nguyen et al. 2015; Zhang et al. 2020). The phylogenetic analysis revealed that tribe Maleae containing genera *Malus*, *Chaenomeles*, *Sorbus*, *Photinia*, *Pyrus*, *Eriobotrya*, *Crataegus*, and *Amelanchier* was strongly supported as monophyletic, and the genus *Crataegus* L. was sister to the genus *Amelanchier* Medik.. The cpDNA of *C*. *kansuensis* is closely related to that of *C. chungtienensis* and *C. marshallii*. The complete chloroplast genome reported in this study will be a valuable resource for future studies on genetic diversity, taxonomy, and phylogeny of family Rosaceae.

**Figure 1.**
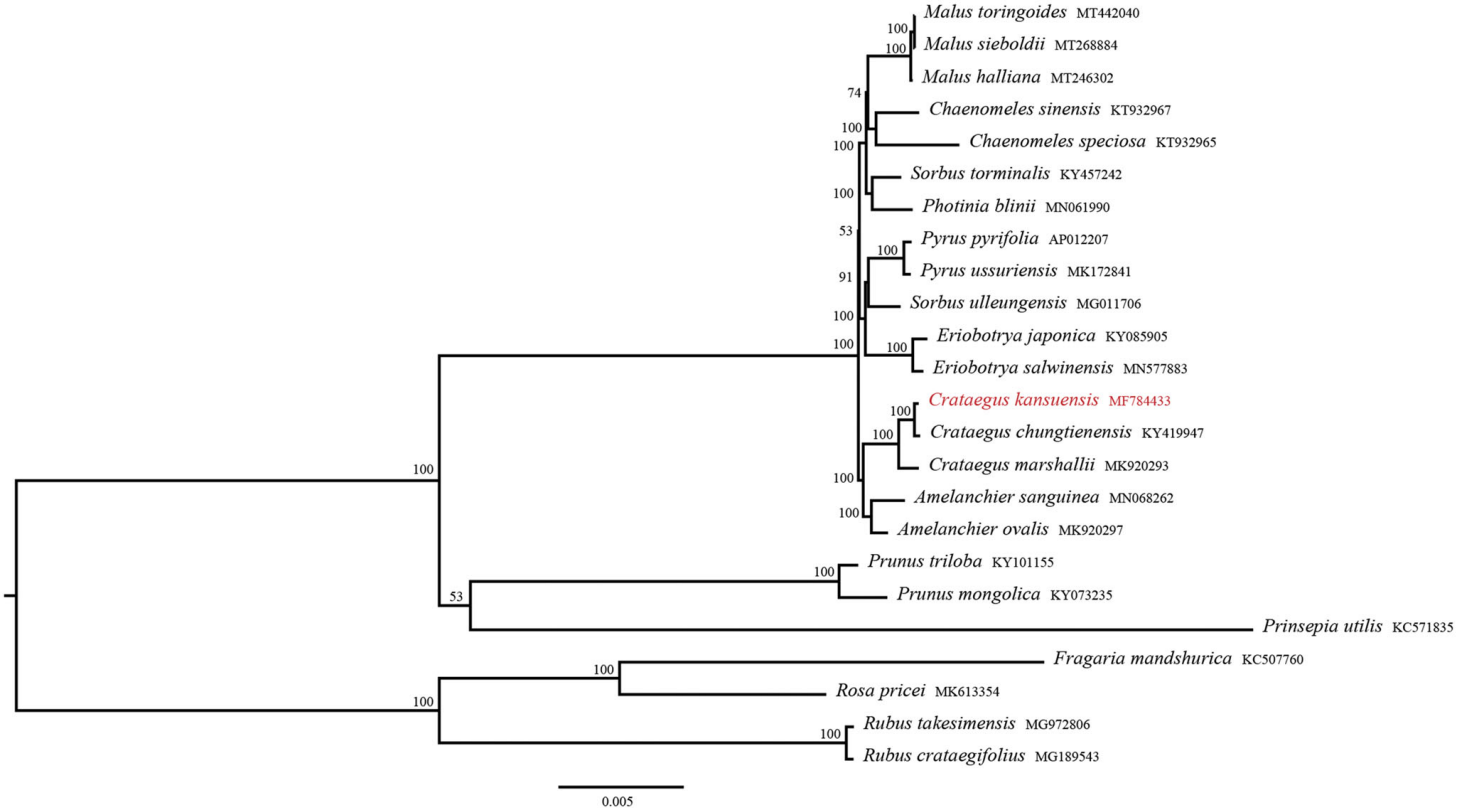
Phylogenetic tree reconstruction of 24 taxa using maximum-likelihood (ML) methods based on 77 genes in the chloroplast genome sequences. ML bootstrap support value presented at each node.

## Data Availability

The data that support the findings of this study are openly available in GenBank of NCBI at https://www.ncbi.nlm.nih.gov/nuccore/MF784433.1/, reference number MF784433.

## References

[CIT0001] Bankevich A, Nurk S, Antipov D, Gurevich AA, Dvorkin M, Kulikov AS, Lesin VM, Nikolenko SI, Pham S, Prjibelski AD, et al. 2012. SPAdes: a new genome assembly algorithm and its applications to single-cell sequencing. J Comput Biol. 19(5):455–477. 22506599 10.1089/cmb.2012.0021 PMC3342519

[CIT0002] Dong W, Xu C, Li W, Xie X, Lu Y, Liu Y, Jin X, Suo Z. 2017. Phylogenetic resolution in juglans based on complete chloroplast genomes and nuclear DNA sequences. Front Plant Sci. 8:1148. 28713409 10.3389/fpls.2017.01148 PMC5492656

[CIT0003] Edwards JE, Brown PN, Talent N, Dickinson TA, Shipley PR. 2012. A review of the chemistry of the genus Crataegus. Phytochemistry. 79:5–26. 22608128 10.1016/j.phytochem.2012.04.006

[CIT0005] Li JL, Wang S, Jing Y, Wang L, Zhou SL. 2013. A modified CTAB protocol for plant DNA extraction. Chin Bull Bot. 48(1):72–78.

[CIT0006] Nguyen LT, Schmidt HA, von Haeseler A, Minh BQ. 2015. IQ-TREE: a fast and effective stochastic algorithm for estimating maximum-likelihood phylogenies. Mol Biol Evol. 32(1):268–274. 25371430 10.1093/molbev/msu300 PMC4271533

[CIT0008] Wyman SK, Jansen RK, Boore JL. 2004. Automatic annotation of organellar genomes with DOGMA. Bioinformatics. 20(17):3252–3255. 15180927 10.1093/bioinformatics/bth352

[CIT0009] Zhang D, Gao F, Jakovlic I, Zou H, Zhang J, Li WX, Wang GT. 2020. PhyloSuite: an integrated and scalable desktop platform for streamlined molecular sequence data management and evolutionary phylogenetics studies. Mol Ecol Resour. 20(1):348–355. 31599058 10.1111/1755-0998.13096

